# Research Trends and Structural Characteristics of Healthcare Research in Japan, Including the First Half of the New Coronavirus Spread Period: A Bibliometric Analysis

**DOI:** 10.7759/cureus.84197

**Published:** 2025-05-15

**Authors:** Yasutoshi Moteki

**Affiliations:** 1 Faculty of Policy Studies, Nanzan University, Nagoya, JPN

**Keywords:** academic structure, bibliometrics analysis, japan, research trends, thematic map

## Abstract

This study aims to conduct a bibliometric analysis to characterize the trends and research features of health administration in Japan, in terms of themes and structural aspects, such as institutional affiliations, up to the early stages of the spread of the new coronavirus. Literature data were obtained from the United States National Institutes of Health (NIH) database, using the search formula (Healthcare[Title/Abstract] AND Japan[Title]), and the dataset was obtained on March 15, 2025. The total number of data points analyzed was 1066. Research trends, such as the characteristics of themes based on KeyWords Plus (Clarivate, Philadelphia, USA) and their changes, and the academic structure focusing on the country of origin, institutional affiliations, and publication journals, were quantitatively analyzed using the bibliometrics tools in the R package (Biblioshiney interface) for literature up to 2021, when the impact of coronavirus disease of 2019 (COVID-19) became pronounced in Japan.

The KeyWords Plus analysis revealed a substantial research emphasis on healthcare human resources, and confirmed an increase in COVID-19-related research around 2020, when the impact of the novel coronavirus infection spread in Japan. Notably, the results of the bibliometric analysis highlight the aspect that healthcare human resources was one of the main focuses of the study area. However, limitations of textmining methods were observed in the export function of the CiNii database (National Institute of Informatics, Tokyo, Japan), which comprehensively collects articles written in Japanese. In order to grasp research trends in the field of healthcare in Japan, regardless of language, it is necessary to enhance multilingual support in Japanese academic information databases and develop an international academic information database (such as Web of Science, Scopus, OpenAlex, etc.) to expand the scope of collection.

## Introduction and background

Coronavirus disease of 2019 (COVID-19) has had a significant impact on healthcare management in developed countries, including Japan. On Monday, May 8, 2023, new coronavirus infections in Japan were assigned a legal status of category 5 with a relatively low degree of countermeasures. Until that time, the number of deaths from this infection in Japan was 74,694. The impact on Japan’s medical policy, medical institutions, and the lives of Japanese over the last 5 years has been enormous [1]. This disruption has also substantially impacted research trends in healthcare administration. Recent literature [2-4] has extensively examined the impact of COVID-19 on specific areas of medicine through systematic and meta-analyses approaches. The impact of COVID-19 on the healthcare sector is significant, and the negative impact on the (potential) medical workforce has been variously discussed. Kitayama [5] showed in a textual analysis of social networking sites that the expansion of COVID-19 caused anxiety and other negative feelings among examinees about the national examination for pharmacists, one of the healthcare-related professionals, in Japan. 

In recent years, literature review methodologies such as systematic analysis, meta-analysis, scoping review, and bibliometric analysis have been widely used in various fields, including medical research, to reanalyze existing literature, including quantitative methods [6]. South and Rodgers [7] provide a scoping review of health topics published in the journal *Systematic Reviews*. Akhter et al. [8] explain in detail the differences between the methods of each review, especially between systematic reviews and meta-analyses. A meta-analysis typically aggregates findings from studies using consistent methodologies (e.g., tests of differences in means) to assess effects of a specific treatment or intervention or a particular research question, offering a higher level of evidence than individual randomized controlled trials (RCTs). A systematic review collects articles on a specific topic from multiple article databases, such as PubMed and Web of Science, for a specific period of time, narrowing the literature down to approximately 20 to 30 articles under certain conditions, summarizing the characteristics of each individual article, and then targeting the results of a series of reviews.

Our bibliometric analysis is closer to a systematic analysis; however, it is simpler and more exploratory, focusing on the frequency of bibliometric data and words used, without extensively narrowing the literature. This review is intended to provide an overview of research methods and results in the research field and to clarify points that need to be addressed in future research. Preferred Reporting Items for Systematic Reviews and Meta-Analyses (PRISMA) clarifies the procedures to be followed for these two review methods, with a flow chart detailing the refinement process and review method. Currently, the PRISMA 2020 statement [9] is widely followed for meta-analyses, ensuring a certain degree of reproducibility of review studies.

Scoping reviews are among the most recent review methodologies, and their application is rapidly increasing. The article by Mak and Thomas [10] provides an introductory explanation on how to perform a scoping review, aimed mainly at young researchers in the United States. Our bibliometric analysis is also similar to a scoping review. The latter involves narrowing down the literature to about 30 references, followed by a qualitative introduction and evaluation of individual references to elucidate the current state of the field in question. Bibliometric analysis does not involve such a narrowing down of literature, and it analyzes the research in the field in question. Bibliometric analysis is a more exploratory method that does not extensively restrict the literature and tries to grasp the current state of research in the field. Scoping reviews are similar to systematic reviews as they also involve collecting data from article databases and narrowing the list of articles to be analyzed. However, the results of the statistical analysis are compiled and integrated in a systematic review, but not in a scoping review.

A scoping review will eventually restrict the list to approximately 20 to 30 articles (exclusively open-access (OA) articles), each of which will be reviewed individually, and will conclude with a discussion of the current state of the research field under analysis and identify gaps for future investigation. Scoping reviews are the most exploratory of the review methods listed thus far. They are often conducted before systematic reviews or original studies, such as RCTs, to gain an overview of the existing literature in the field. Because scoping reviews are based on article databases, bibliometric information - derived from article metadata - is often used in the early phases of analysis. As described above, the bibliometric analysis in the current study is similar to a scoping review in terms of the procedure, focusing exclusively on bibliographic information and analyzing the characteristics of research trends and academic structures (research structure), including authors, affiliated institutions, affiliated countries, their networks, and the relationships among them [11].

This study also employed bibliometric analysis using the PubMed database as the data source. The author compared the results of a text-mining analysis of article titles in Japanese academic journals with those of the period before and after the spread of the new coronavirus infection in Japan, and found commonalities between the two methods as well as differences that could be detected only by bibliometric analysis. The results were compared with the author's text-mining analysis of article titles in Japanese academic journals [12]. Similar to the author, Sakai et al. [13] attempted to analyze research trends by text mining analysis of article titles in a specific field (occupational medicine) in health care. A study of antimicrobial stewardship conducted by Japanese pharmacists using the same method of bibliometric analysis as in the author's study, targeting research in the medical field, is available [14]. The present study is distinguished by its unique approach, involving a comparative analysis of the outcomes derived from text mining of article titles and those obtained from bibliometric analysis of articles on research trends in the domain of healthcare in Japan during the same period, encompassing the era of the increase in the novel coronavirus infection. The present article was posted on the SSRN preprint server on May 5, 2025.

## Review

Data extraction and search approach

**Figure 1:**
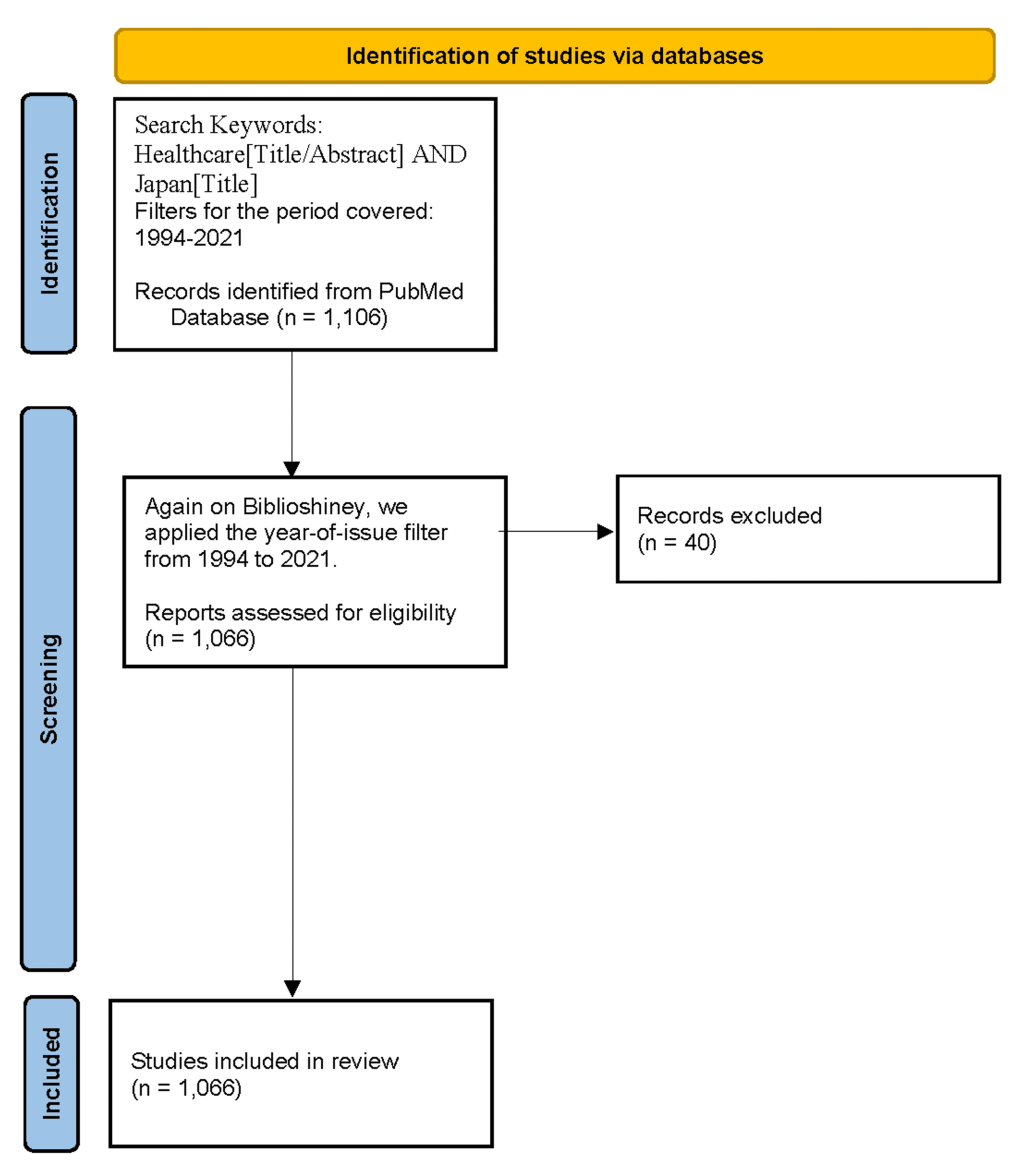
Flowchart for obtaining and filtering literature according to PRISMA 2020 PRISMA: Preferred Reporting Items for Systematic Reviews and Meta-Analyses

To understand the trends and research focus (in terms of themes and structural aspects such as institutional affiliations) of Japanese health administration research up to the time period of the spread of the new coronavirus infection, the author conducted a bibliometric analysis using the PubMed database. The analysis was performed using R software (version 4.5.0; R Foundation for Statistical Computing, Vienna, Austria) with the Bibliometrix package (version 4.3.3), including the Biblioshiny function [15]. The database used was obtained from the United States National Institutes of Health (NIH). The search formula used was: (Healthcare[Title/Abstract] AND Japan[Title]). The filter for the period covered was applied from 1994 to 2021. The type of manuscript and the language of the text were left as defaults and not filtered out. This search yielded 1,106 matches. Similarly, we applied the year-of-issue filter from 1994 to 2021 on Biblioshiny. The total number of data points analyzed was 1,066. A flowchart was created in accordance with the PRISMA 2020 guidelines for obtaining search entries in the database and narrowing down the target data (Figure 1) [16].

Literature data for the analysis were obtained on March 15, 2025. Research trends, such as the characteristics of themes based on KeyWords Plus (Clarivate, Philadelphia, USA) and their changes, and the academic structure focusing on the country of origin, institutional affiliations, and journals were quantitatively analyzed for literature up to the year 2021, when the impact of COVID-19 became pronounced in Japan. Although the checklist was not used to conduct the current research, a prototype checklist is proposed, focusing on items to be included in the article from the viewpoint of reproducibility regarding bibliometric analysis [17]. As the checklist includes, bibliometric analysis has method-specific limitations, which must be borne in mind when interpreting the present study.

Descriptive analysis

Table 1 presents the dataset summary/descriptive statistics of the target articles. KeyWords Plus is a set of keywords converted into general higher-level concepts, separate from the author-provided keywords. In PubMed, KeyWords Plus are often reflected as MeSH terms (ID), while author keywords (DE) appear separately. However, when data are exported from PubMed in CSV format, author keywords and citation information are often missing - an issue confirmed by the author in the current dataset. Table 1 provides an understanding of the characteristics of the overall dataset, which will be reviewed in later analyses, including the annual growth of publication volume.

**Table 1:** Dataset summary/descriptive statistics Note: Areas with missing data sets to zero are indicated with dashed lines.

Description	Results
MAIN INFORMATION ABOUT DATA	
Timespan	1994–2021
Sources (Journals, Books, etc.)	458
Documents	1066
Annual Growth Rate (%)	19.06
Document Average Age	9.1
Average citations per document	-
References	-
DOCUMENT CONTENTS	
KeyWords Plus (ID)	3360
AUTHORS	
Authors	3914
Authors of single-authored documents	86
AUTHORS COLLABORATION	
Single-authored documents	100
Co-authors per document	6.01
International co-authorships (%)	14.26

Institutional and structural characteristics

**Figure 2:**
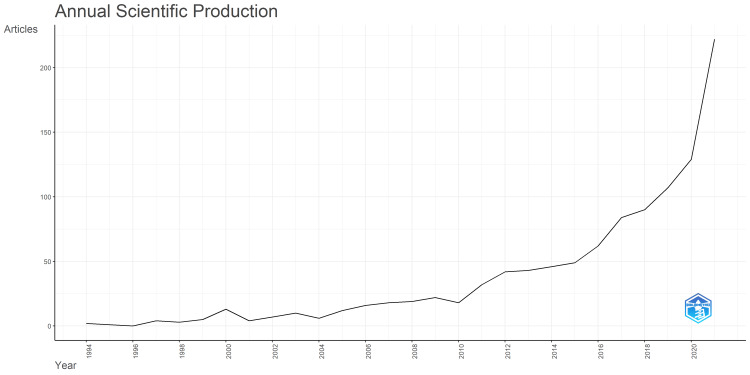
Change in the number of English-language articles on healthcare in Japan indexed in PubMed

Figure 2 illustrates the changes in the number of articles published per year over the study period. The number of publications has been increasing annually, with a notable surge beginning around 2019 when the impact of the new coronavirus appeared worldwide. In 2021, the number of English-language articles related to Japanese healthcare in PubMed surpassed 200 (Figure 2).

**Figure 3:**
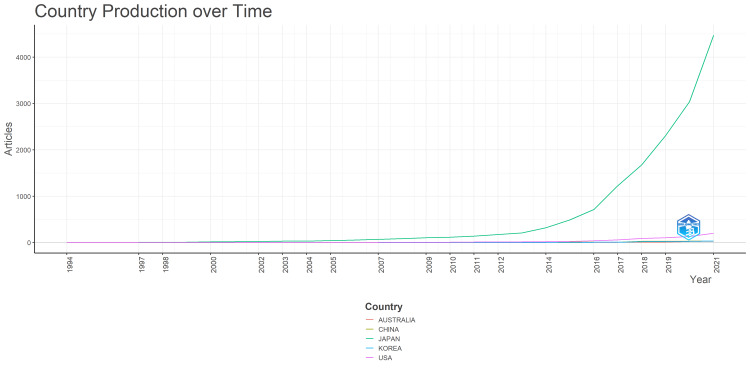
Change in the number of articles by country of the first author's institutional affiliation

Figure 3 shows the change in the publication volume by the country of the first author's institutional affiliation. This figure shows that the number of English-language papers on Japanese healthcare gradually increased around 2016 and showed a marked rise after 2019, influenced by the pandemic. Figure 3 shows that the number of English-language articles in Japan increased shortly before COVID-19. Going by the country of the first author in English, Japan had the highest number of publications, followed consistently by the United States.

**Figure 4:**
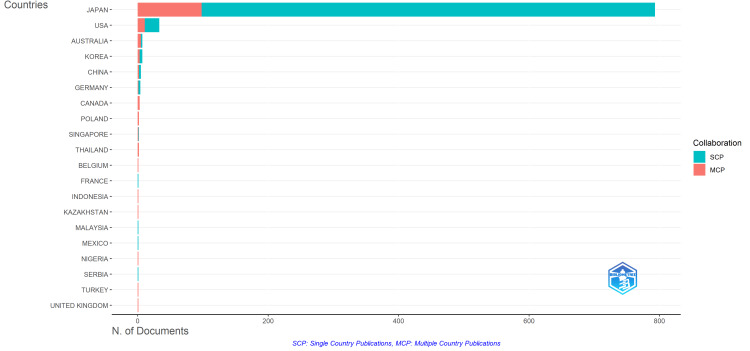
Number of English-language, first-authored articles on healthcare in Japan by country

Figure 4 shows the number of first-authored articles in the field of healthcare by country, distinguishing between single-country authored (SCP; shown in blue) and multi-country co-authored (MCP; shown in orange) articles. Due to limitations in PubMed’s CSV export format, which omits corresponding author information, institutional affiliation was determined based on the first author. The same is true for all subsequent analyses. This figure shows that in Japanese institutions, research in the field of healthcare has exclusively been published as single-country co-authored papers.

**Figure 5:**
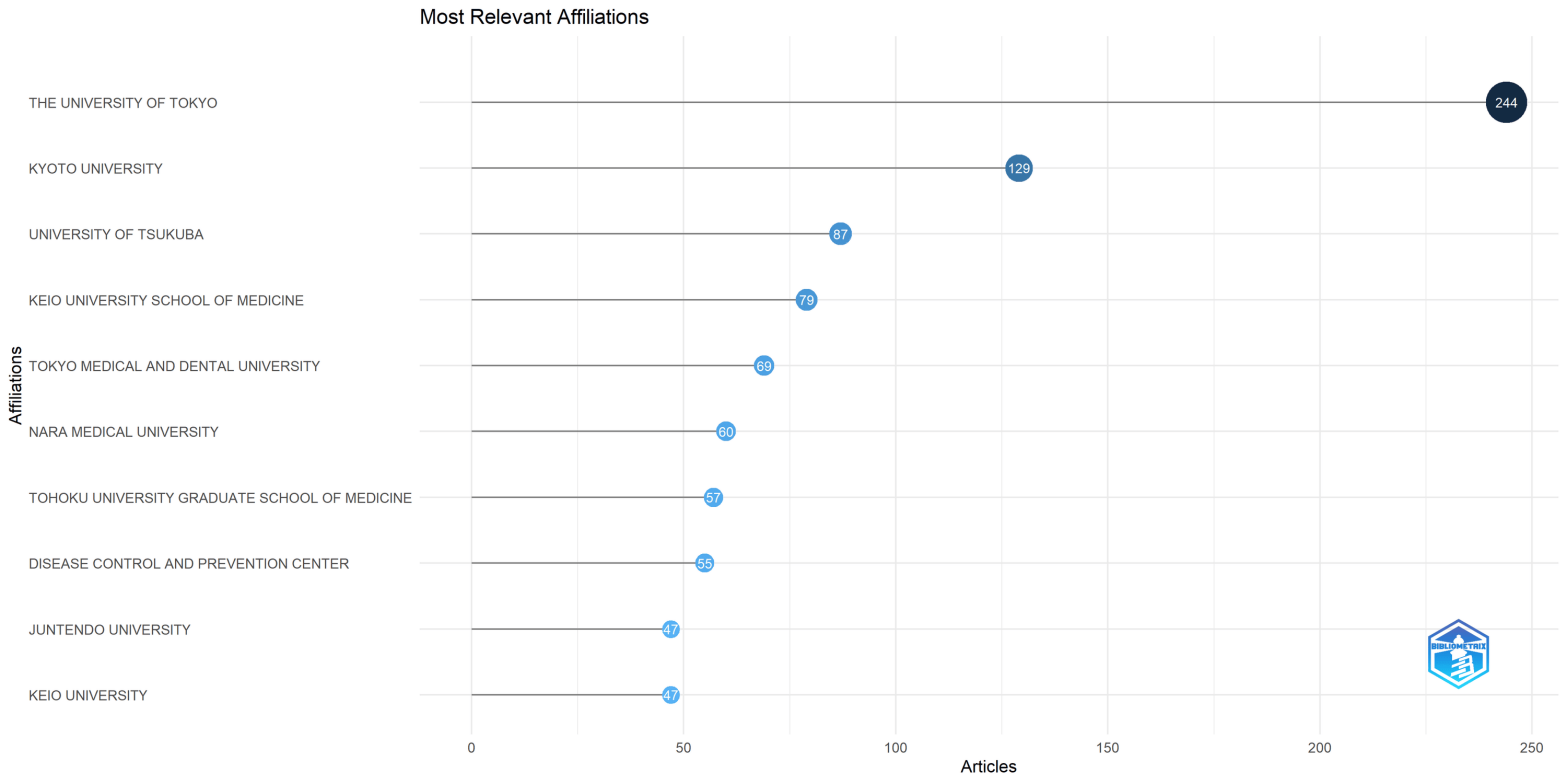
Institutional affiliations of the first author for the target articles

**Figure 6:**
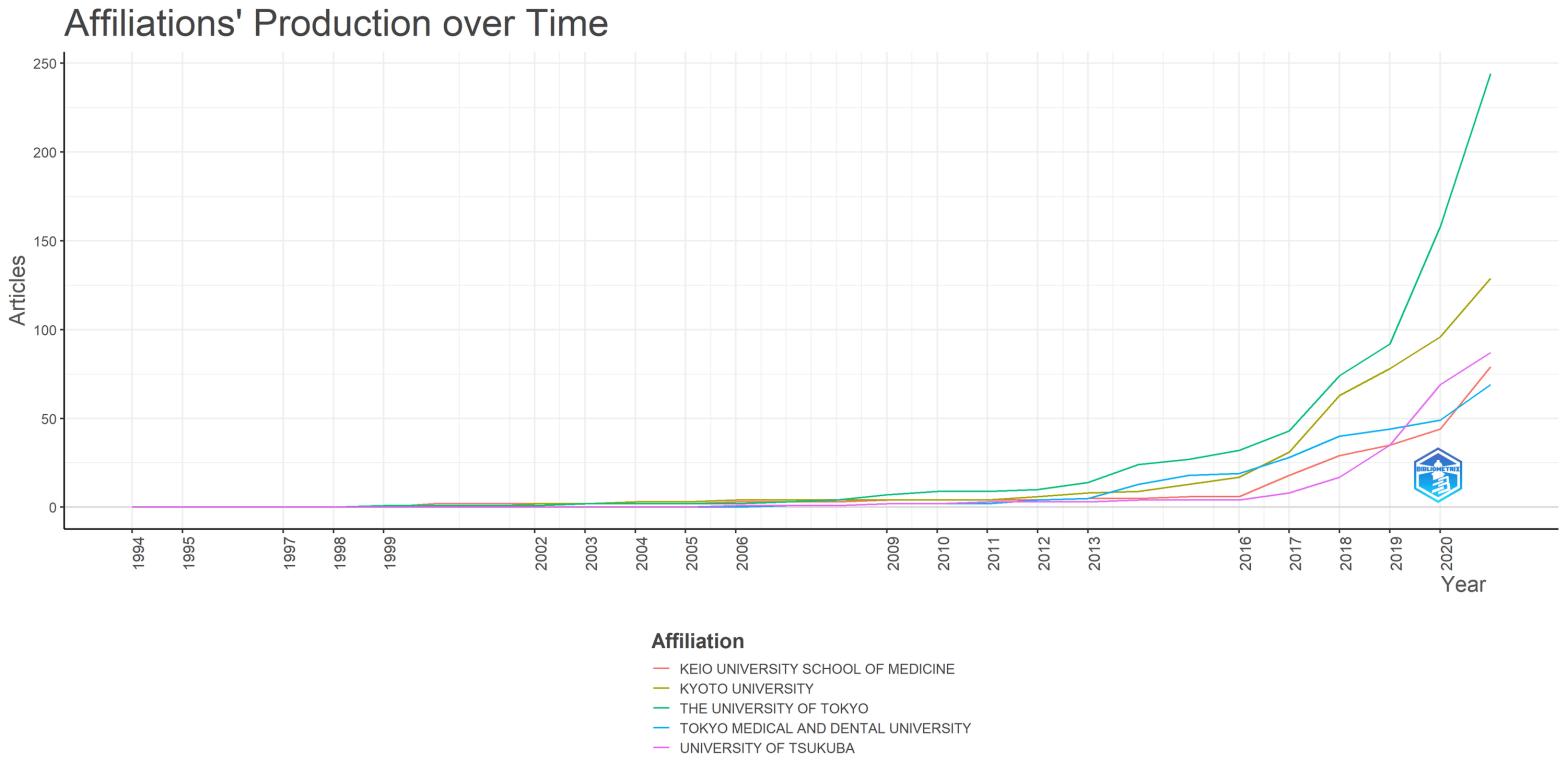
Temporal change in the number of papers by the first author's institutional affiliation

The authors' institutional affiliations are summarized in Figure 5, which displays the institutional affiliations of the first authors of the target articles. From first to last are the University of Tokyo, Kyoto University, and the University of Tsukuba, with a nearly two-fold difference in the number of publications between first and second places. The Bibliometrix package used for the tabulations handles the inclusion or exclusion of affiliated hospitals, so that the same university may be recorded separately. In a related issue, we noticed inconsistent processing of institution names ending with a period, causing the same institution to be recorded separately. Therefore, based on a list of all institutions (in Excel), the source data was preprocessed by removing periods for institutions appearing at least 10 times. The subsequent dataset was used throughout the study. Figure 6 shows the change in the number of papers published by the first author’s institutional affiliation over time. The University of Tokyo has consistently ranked first since 2009. Notably, the gap between the first and second places widened significantly after 2019, probably due to an increase in research related to novel coronaviruses.

**Figure 7:**
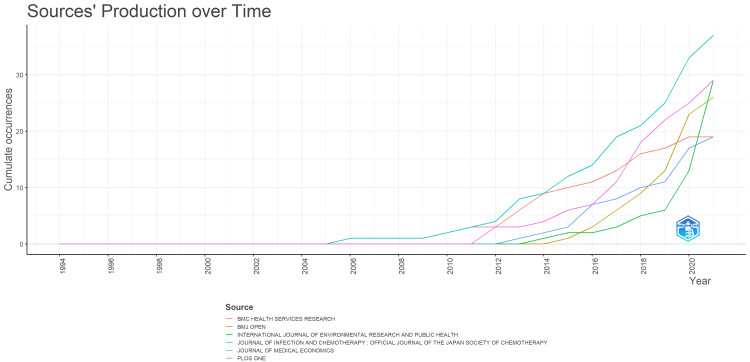
Temporal change in the journals publishing the target articles

Figure 7 shows the distribution of target articles by journal over time. The *Journal of Infection and Chemotherapy*, the official journal of the Japanese Society of Chemotherapy, the Japanese Association for Infectious Diseases, and the Japanese Society for Infection Prevention and Control, was ranked first in publication frequency, including during the spread of the new coronavirus infection. This journal was published by three Japanese academic societies, including Nihon Kansensyo Gakkai (the Japanese Association for Infectious Diseases), in cooperation with Elsevier. The second-ranked journal, represented in pink in Figure 7, is *PLOS One*, an international OA journal whose contributions have increased since around 2016, including during the spread of the novel coronavirus infection. *BMJ Open*, an open-access journal affiliated with the British Medical Association and represented in ochre, ranked third in 2020. In 2021, *BMJ Open *had fallen to fourth place in the overall ranking.

Thematic analysis using KeyWords Plus

Finally, we analyzed the research themes of the target papers using keywords and bibliometric data. The content of this section overlaps with the aim of the author's previous study [12], which analyzed changes in research themes over time, focusing on the title of the article. Differences between the two methods are discussed in the following Discussion section.

**Figure 8:**
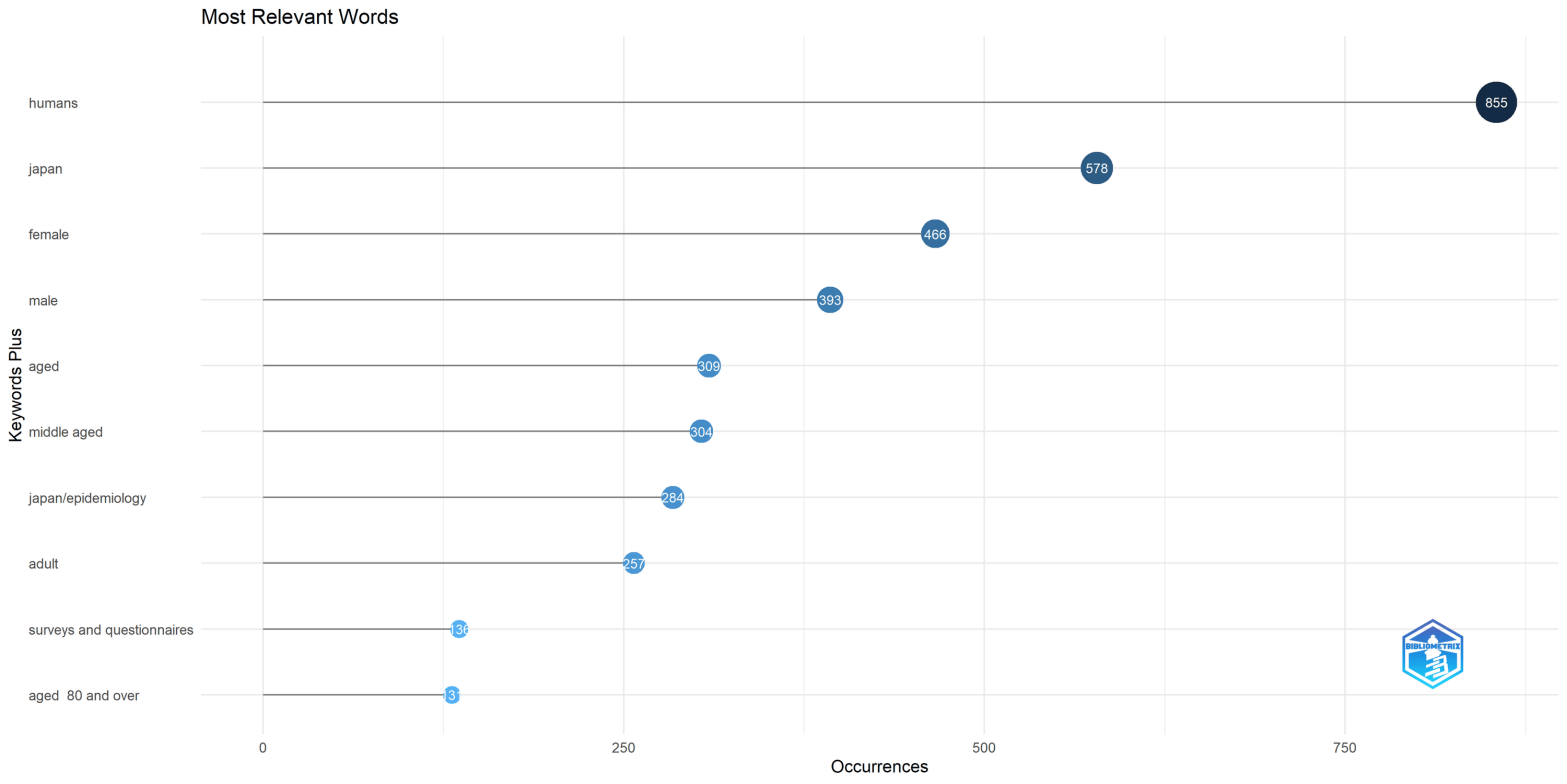
Most frequently expressed words with respect to Keyword Plus

Figure 8 presents the most frequently expressed words with respect to KeyWords Plus. The most common terms include humans, Japan, female, male, aged, and middle-aged, many of which seem to be related to the population being analyzed or considered.

**Figure 9:**
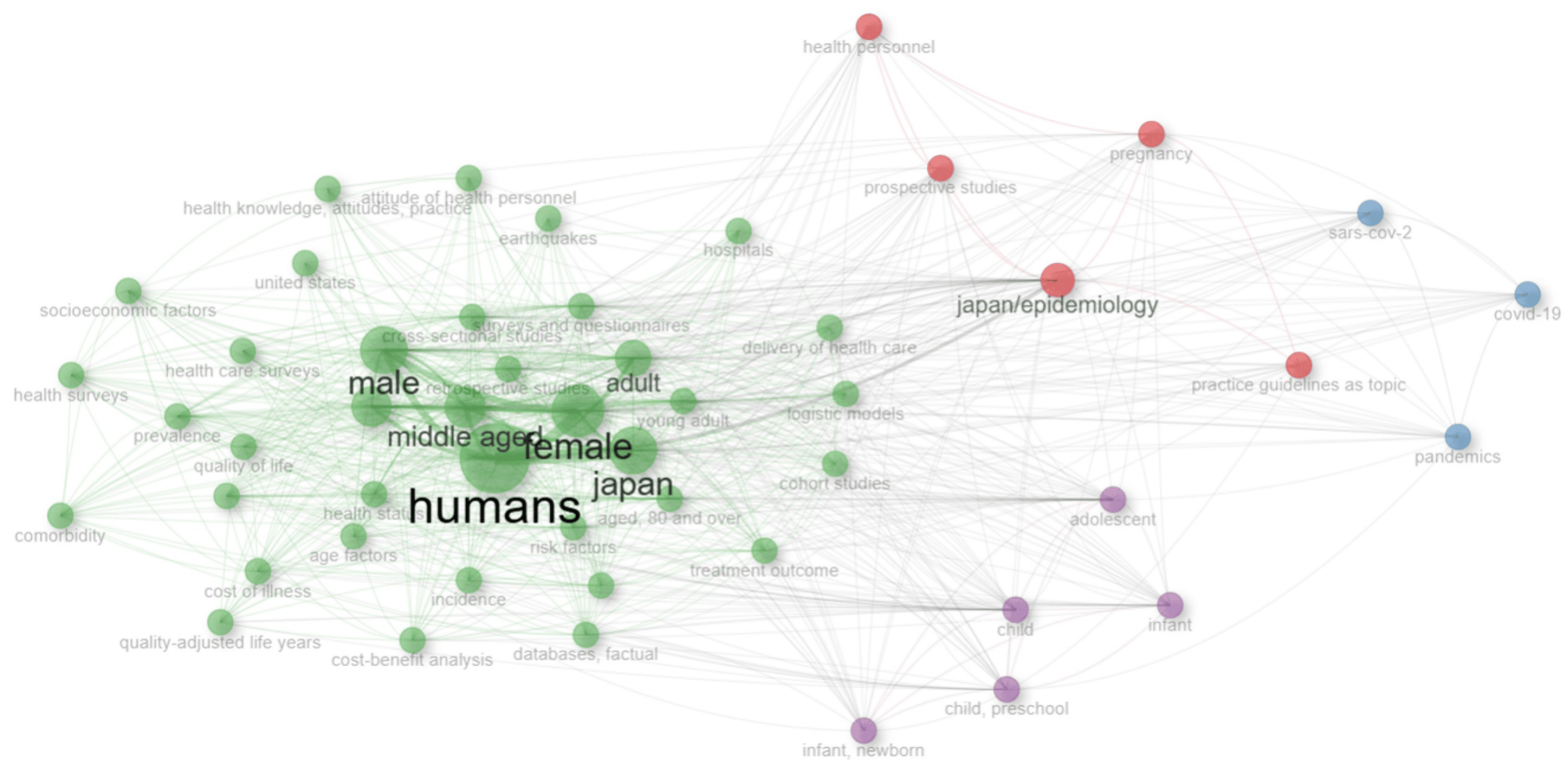
KeyWords Plus co-occurrence network

Figure 9 shows the keyword and co-occurrence networks. Lines are drawn between frequent simultaneous occurrences in the bibliographic information of the same article. Words that are high in frequency are represented in bold. The top KeyWords Plus terms, having the highest frequency, are represented in green on the left side of the figure. Another group worth noting is the group represented in purple, which comprises the words related to juveniles, such as children, infants, and adolescents, which frequently co-occur with each other.

**Figure 10:**
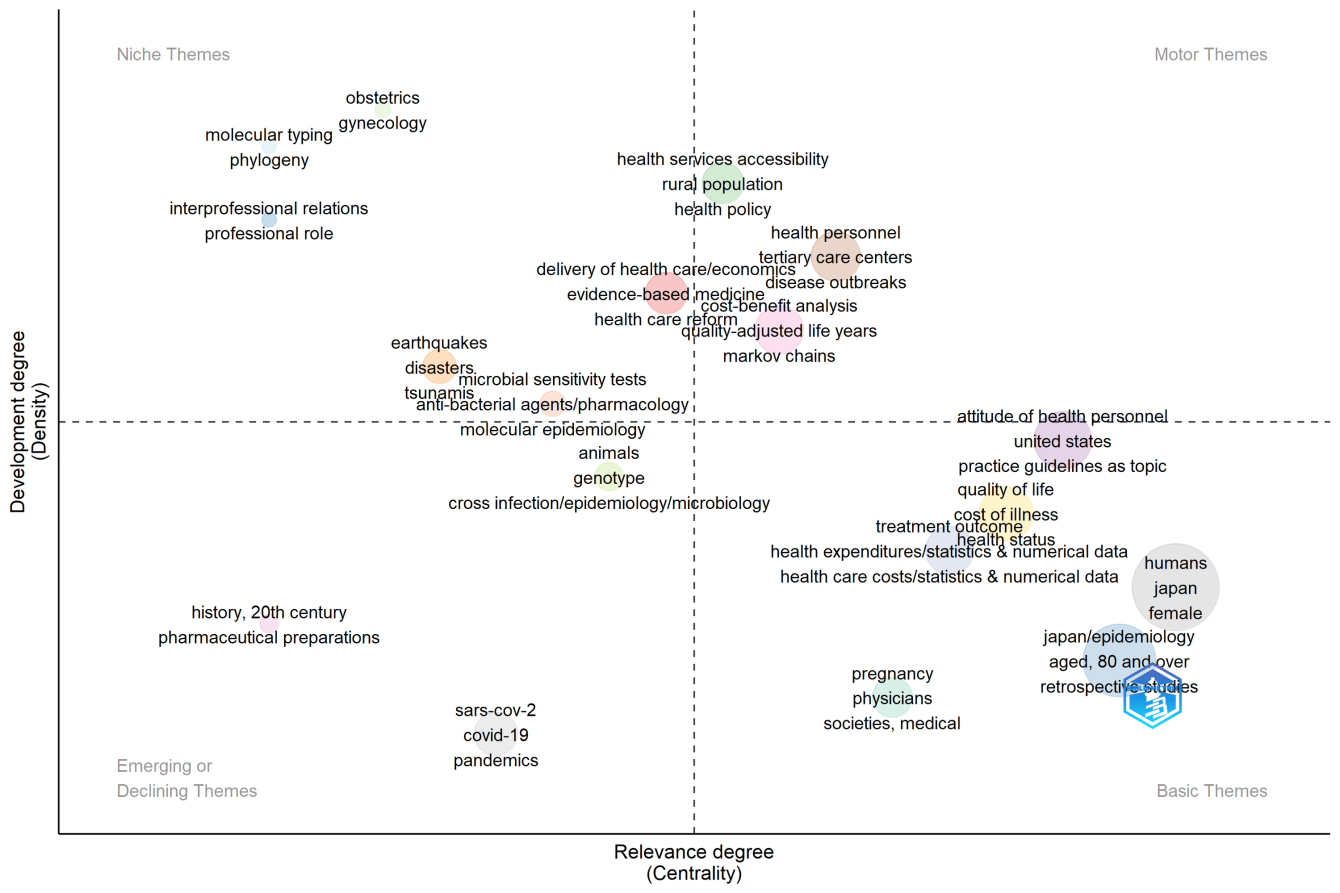
Thematic map of KeyWords Plus

Figure 10 shows the thematic map of KeyWords Plus. Thematic maps are used in bibliometrics to visually represent major themes and their relationships within a particular research area. It is typically created based on the results of co-authorship network analysis, co-citation analysis, and keyword co-occurrence network analysis. A thematic map is a two-dimensional diagram that categorizes research themes into four quadrants based on two indices: Centrality and Density. Centrality indicates the importance of a theme. This indicator measures how strongly keywords related to a theme are connected to other themes in the entire research field (external relevance). Themes with high centrality played a fundamental role in this research. Density indicates the degree of theme development. It measures the strength of the ties (internal relevance) between keywords related to a theme. A high-density theme is considered to be well-discussed and developed internally. Based on these two axes, words can be categorized and illustrated as Motor Themes, Basic Themes, Niche Themes, and Emerging or Declining Themes.

The label size was changed to 0.15 to avoid an overlap of the KeyWords Plus labels to be displayed. Other than that, the default settings were used, that is, the number of words was 250, the number of labels was 3, community repulsion was 0, min cluster frequency per thousand docs was 5, and the clustering algorithm was Walktrap. Infectious disease-related keywords such as SARS-CoV-2, COVID-19, and pandemics are represented in the lower left quadrant, indicating low centrality and density, and that they are new and growing research topics, as will be shown later in this analysis. Conversely, more centralized and denser themes are shown in the upper-right quadrant. One such theme group is represented by a dark brown circle that includes the Health Personnel keyword. Such themes related to human resources reflect their foundational role in healthcare research. This finding is consistent with that of a previous study [12] analyzing a single healthcare management journal in Japan.

**Figure 11:**
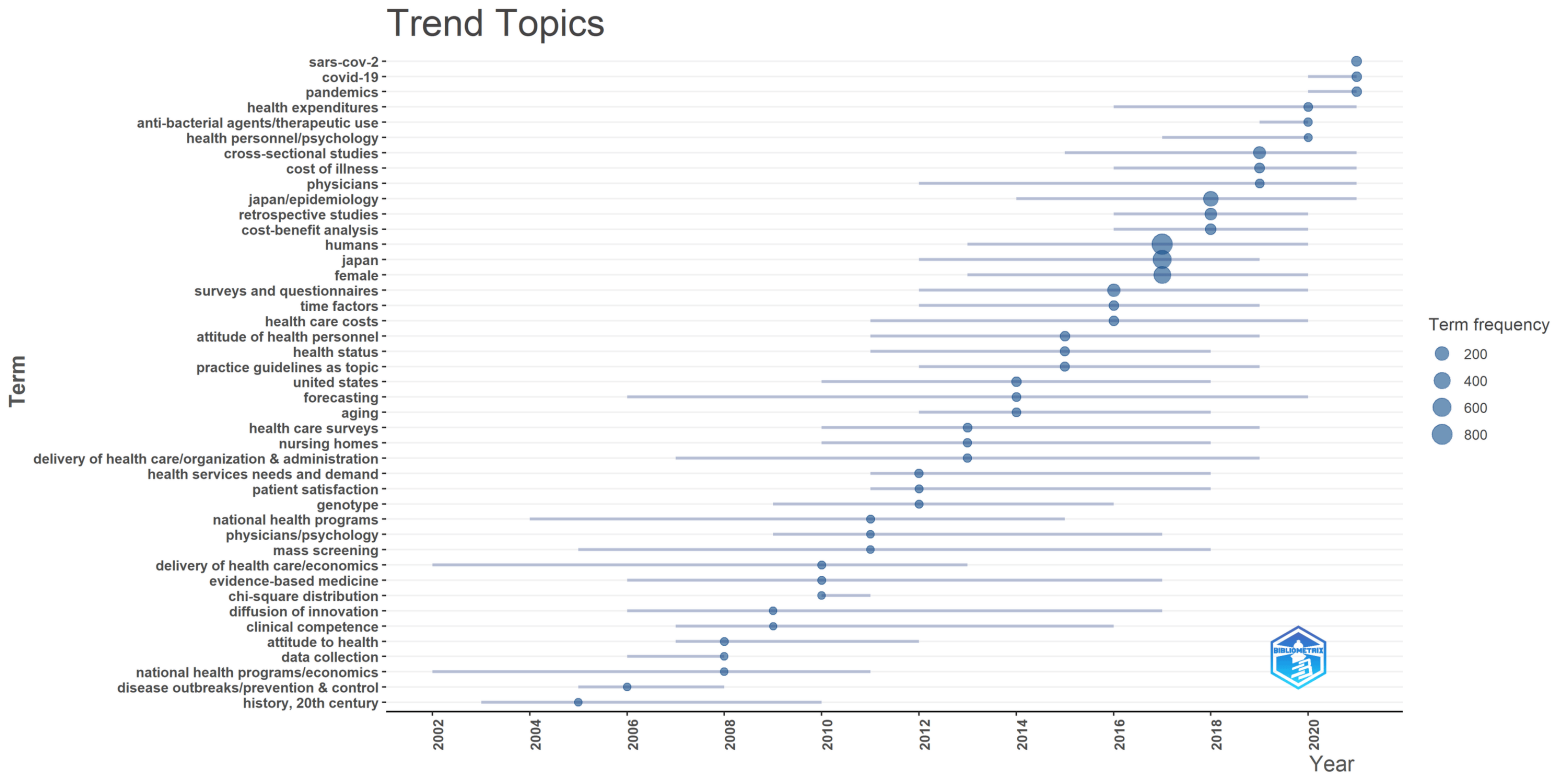
Year-by-year evolution of characteristic themes based on KeyWords Plus

Figure 11 shows the evolution of the characteristic themes from year to year. The three novel coronavirus infection-related keywords, namely SARS-CoV-2, COVID-19, and pandemics, represent the characteristic keywords for the years 2020-2021, especially 2021.

Discussion

This study presents a quantitative analysis of research in the healthcare field in Japan using bibliographic data from the PubMed database. Regarding the evolution of research themes, our findings are consistent with those from the author's previous text-mining analysis of article titles [12], particularly the emphasis on topics related to healthcare human resources. However, this bibliometric analysis provided additional trends related to the research structure - as shown in Figures 4-7, i.e., the authors' institutional affiliations, countries, and journals in which they appear - which is not possible through title-based text analysis alone.

The analysis revealed a notable increase in the number of international English-language publications on Japanese healthcare, particularly those addressing COVID-19, reflecting the global collaborative effort in response to the pandemic. Concurrently, a significant emphasis and growth in research focused on healthcare human resources, underscoring its foundational importance in the field. The observed increase in the number of international English-language papers aligns with the broader global academic trends toward increased international collaboration and dissemination of research findings in English. These trends collectively indicate Japan’s increased integration into the global healthcare research landscape during this period.

The author's study applies content analysis using only the text data of article titles [12]. Many healthcare articles written in Japanese can only be searched in the CiNii database (National Institute of Informatics, Tokyo, Japan), meaning that although the method is limited to text mining analysis of article titles, the significance of using CiNii for research is not lost. In other words, the current specification of the CiNii database has significant limitations when performing bibliometric analysis, and text mining analysis of article titles remains effective for analysis of trends in medical research on Japan written in Japanese. The author’s study was a bibliometric analysis of articles on Japanese healthcare written primarily in English. In this analysis, we reaffirmed the importance of international OA journals as a forum for the publication of such research, as the number of research articles written in English by Japanese scholars has rapidly expanded during global public health crises.

## Conclusions

We conducted a bibliometric analysis to characterize trends and research focus (in terms of themes and structural aspects, such as institutional affiliations) in Japanese health administration research until just before the spread of the new coronavirus infection. Literature data were retrieved from the NIH database using the search formula (Healthcare[Title/Abstract] AND Japan[Title]). The data for the analysis were obtained on March 15, 2025. The total number of data points analyzed was 1066. Research trends, such as the characteristics of themes based on KeyWords Plus and their changes, and the academic structure focusing on the country of origin, institutional affiliations, and journals were quantitatively analyzed up to 2021, when the impact of COVID-19 spread in Japan was prominent, using the bibliometrics of the R package (Biblioshiny interface).

An important finding of this bibliometric analysis is the distinction between traditional and emerging themes in the Japanese healthcare sector. A substantial focus on healthcare human resources was evident through the KeyWords Plus analysis. Additionally, the study confirmed a sharp increase in COVID-19-related research around 2020, when the impact of the novel coronavirus infection spread in Japan. A key limitation of textmining methods lies in the export function of the CiNii database, which comprehensively collects articles written in Japanese. In order to grasp research trends in the field of healthcare in Japan, regardless of language, it is necessary to enhance multilingual support of Japanese academic information databases, develop an international academic information database (like Web of Science, Scopus, OpenAlex, etc.), and expand the scope of collection.
